# Ganglioside enhances the immunogenicity of nanoparticles displaying short synthetic tumor neoepitopes and epitopes

**DOI:** 10.7150/thno.128187

**Published:** 2026-03-17

**Authors:** Shiqi Zhou, Yuan Luo, Maarten K Nijen Twilhaar, Wei-Chiao Huang, Amal Seffouh, Yiting Song, Yafei Su, Breandan Quinn, Yun Wu, Sriram Neelamegham, Joaquin Ortega, Joke M.M. den Haan, Jonathan F Lovell

**Affiliations:** 1 Department of Biomedical Engineering. University at Buffalo, State University of New York, Buffalo, New York 14260, United States.; 2 Department of Molecular Cell Biology and Immunology, Cancer Center Amsterdam, Cancer Biology and Immunology, Amsterdam Institute for Infection and Immunity, Cancer Immunology, Amsterdam University Medical Center, Vrije Universiteit Amsterdam, Amsterdam, the Netherlands.; 3 McGill University, Montreal, QC, Canada.; 4 Department of Chemical and Biological Engineering, University at Buffalo, State University of New York, 906 Furnas Hall, Buffalo, NY 14260, United States.

**Keywords:** short peptide, liposomes, vaccine, ganglioside, cancer, CD8^+^ T cell, immunotherapy

## Abstract

**Rationale:**

Short major histocompatibility class I epitopes can be coupled to the surface of immunogenic liposomes to elicit antigen-specific CD8^+^ T cells with vaccination. Here, we examined whether the co-incorporation of GM3 ganglioside, a lipid which targets CD169, a sialic acid-binding receptor, would further improve the immunogenicity of this approach.

**Methods:**

Liposomes were formed with GM3 incorporated and were assessed for CD169 targeting and anti-tumor immunogenicity in murine models. Antigen-specific CD8^+^ T cell populations and characteristics were investigated to examine the effect of liposome formulations.

**Results:**

GM3 was readily incorporated into immunogenic liposomes and did not interfere with the particle formation with a recently discovered murine renal carcinoma MHC-I neoepitope, Nes2LR. GM3 enhanced the CD169 targeting of liposomes. Immunization with liposomal particles that included GM3 improved antigen-specific CD8^+^ T cell responses for not only the Nes2LR neoepitope, but for other tumor-associated short MHC-I murine tumor epitopes or mimotopes, including E7_49-57_, and the gp70 mimotope AH1-A5. Immunization of the E7 epitope with GM3 particles induced high-frequency E7-specific CD8^+^ T cells and effectively reversed the tumor growth of large, established TC-1 tumors as an immune monotherapy. Immunization of the Nes2LR neoepitope in GM3 particles led to delayed tumor growth of RENCA tumors.

**Conclusion:**

GM3 ganglioside-containing liposomes that display short peptide epitopes and incorporate immunological adjuvants can be used to elicit potent anti-tumor responses in murine models. Further research is required to further assess the translational potential of this approach.

## Introduction

Cancer vaccines represent a promising form of immunotherapy that aims to educate the immune system to destroy tumor cells through antigen-specific responses 1-4. Endogenous tumor-specific antigens, presented through major histocompatibility complex I (MHC-I), are ideal targets for cytotoxic CD8^+^ T cells in immunotherapy 5. However, MHC-I epitopes are short peptides (8-11 amino acids) with limited immunogenicity as cancer vaccine antigens in clinical studies. Insufficient antigen delivery, rapid degradation, and suboptimal activation of antigen-presenting cells (APCs) remain barriers for peptide-based vaccine design 6-8.

Various approaches have been developed to overcome these limitations, including advanced immunogenic antigen screening, optimization of antigen selection, incorporation of immune-stimulatory adjuvants, and development of nanoparticle-based delivery systems to enhance antigen stability and presentation. Liposomal platforms have shown great potential by enabling co-delivery of antigens and adjuvants to immune cells and improving antigen-presenting efficiency. Building on these advances, our laboratory has previously developed a liposomal delivery system containing Cobalt-Porphyrin-phospholipid (CoPoP) for histidine-tagged antigen capture, along with the liposomal vaccine adjuvants such as Phosphorylated HexaAcyl Disaccharide (PHAD) and QS-21, referred to as CPQ liposome 9-12. This platform has been validated with multiple short MHC-I peptide antigens, including tumor-associated, tumor-specific, and mimotope peptides, demonstrating enhanced antigen-specific CD8^+^ T cell responses in various murine tumor models 13-16. Nevertheless, further improvement of peptide vaccine performance may be achieved by directing liposomal vaccines to specific APC subsets to enhance antitumor immune response.

Transmembrane protein CD169, also known as sialoadhesin (Sn) or Siglec-1, is expressed on a subset of macrophages and dendritic cells. Recent studies have identified CD169^+^ (Siglec-1^+^) macrophages as an important immune cell population for antitumor immune responses and effective cross-priming of CD8^+^ T cells 17-22. Incorporation of the sialic acid-containing ganglioside GM3 into liposomal nanoparticles has been shown to promote selective interaction with CD169-expressing cells 23. However, whether natural GM3 gangliosides can be functionally integrated into CoPoP-formulated liposomal vaccines to enhance peptide immunogenicity has not been explored. In this study, we demonstrate that incorporation of bovine milk-derived GM3 into CPQ liposomes promotes preferential interactions with CD169-expressing antigen-presenting cells, enhances antigen-specific immune responses across distinct short peptide antigens, and improves therapeutic efficacy in two tumor models.

## Results

### Characterization of liposomes incorporating gangliosides

**Figure 1:**
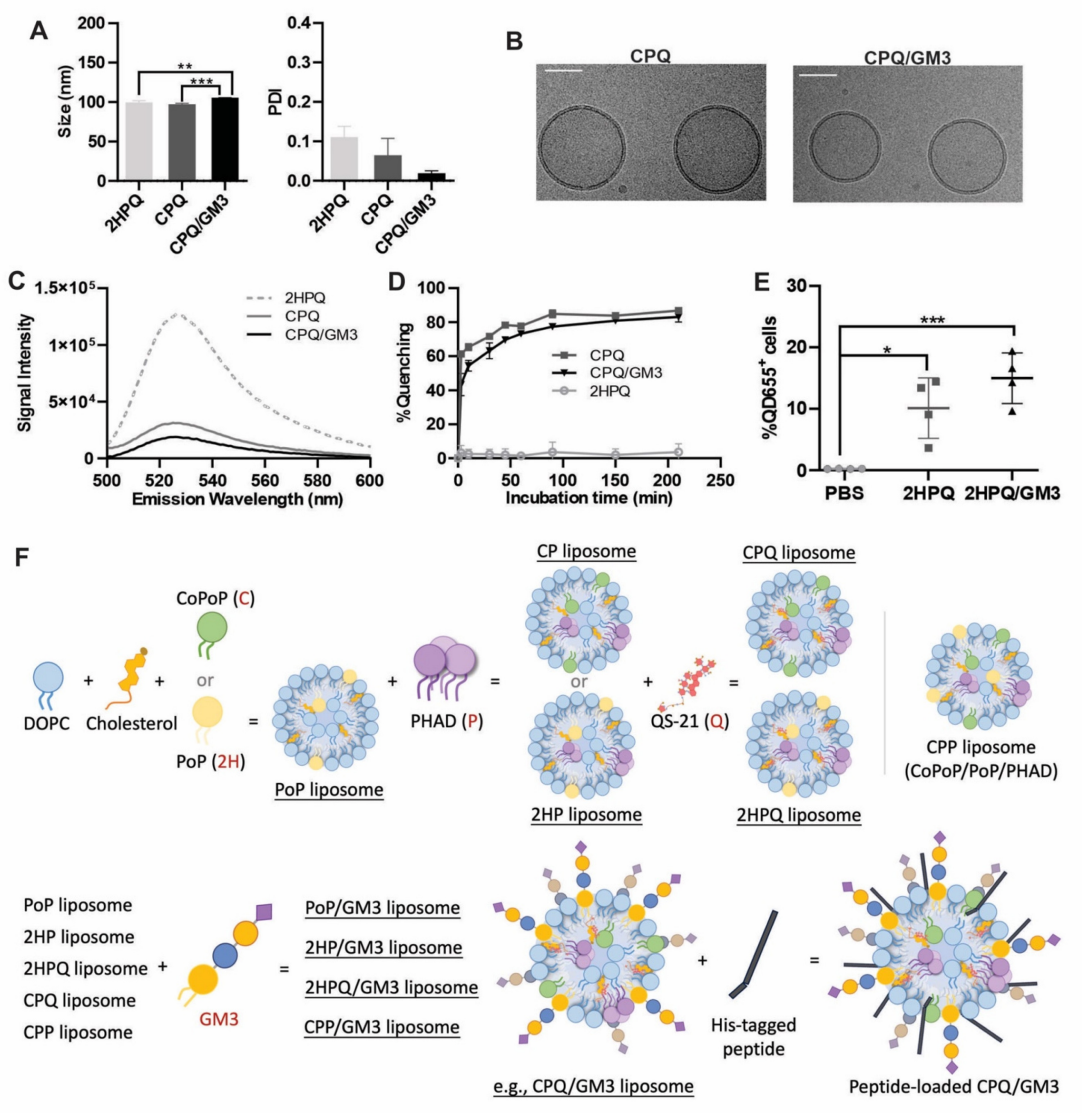
** Characterization of ganglioside GM3 incorporated liposomes. A)** Indicated liposome size from three independent studies. **B)** Cryo-EM images of CPQ liposomes with or without GM3 lipids. The scale bar corresponds to 50 nm.** C)** Signal intensity of fluorescent peptide after incubating with liposomes (n = 3). **D)** Fluorescence quenching (reflecting peptide binding to liposomes) kinetics of HiLyte488-labeled Nes2LR peptide to 2HPQ, CPQ, or CPQ/GM3 liposomes (n = 3). **E)** Percentage of QD655 signal-positive cells in all living cells collected from lymph nodes 24 h post-single vaccination of indicated liposomes. **F)** Schematic diagram of liposome formulations used in this study. Error bars show mean ± std. dev. *, **, ***, and **** indicate *P* ≤ 0.05, 0.01, 0.001, and 0.0001, respectively. A) was analyzed by One-way ANOVA followed by Tukey's multiple comparisons test.

Natural GM3 was incorporated during the formulation of liposomes that contain CoPoP, PHAD, and QS-21. In this study, CoPoP/PHAD/QS-21 liposomes are referred to as CPQ, and CPQ liposomes incorporated with GM3 are referred to as CPQ/GM3. The CPQ liposomes and their identical liposomes lacking cobalt in the CoPoP lipid macrocycle (termed 2HPQ liposomes, since they contain two hydrogens instead) had a size of around 100 nm (**Figure 1A**). The incorporation of bovine milk-extracted natural GM3 into CPQ liposomes resulted in a slightly larger size of 105 nm. All liposomes exhibited a polydispersity index less than 0.2, consistent with a reasonably monodisperse particle size population. The incorporation of GM3 did not change the spherical morphology nor the unilamellar structure of liposomes (**Figure 1B**).

Next, to assess peptide binding to liposomes (mediated by the CoPoP contained within them), a Hilyte488 fluorescently labeled peptide was used to observe resonance energy transfer quenching that occurs upon binding to liposomes 24. The fluorescently labeled Nes2LR neoepitope was incubated with different liposomes, and the fluorescence emission spectra were recorded (**Figure 1C**). Compared to the peptide fluorescence when mixed with PoP liposomes lacking cobalt (2HPQ liposome), the neoepitope became quenched with all formulations containing CoPoP. The fluorescent quenching is caused by fluorescence resonance energy transfer (FRET) between fluorescently labeled peptides and CoPoP lipid. This can be used to reflect the binding of the peptide to liposomes. Based on real-time kinetic measurements, the GM3-modified CPQ liposomes, immediately quenched more than 50% of the fluorescent peptide fluorescence, increasing to approximately 80% after 3 h of incubation (**Figure 1D**). Liposomes lacking cobalt (i.e. 2HPQ) would not be expected to induce any peptide binding, and as expected Nes2LR fluorescence peptide quenching was not observed. These data confirm the robust incorporation of the Nes2LR peptide in cobalt-containing liposomes.

Detecting the safety and potential side effects is important for evaluating GM3-incorporated CPQ liposome vaccines. Serum and complete blood count (CBC) panel tests for BALB/c mice were conducted 7 days after administration of a single vaccination of CPQ liposomes or CPQ/GM3 liposomes. No data reflecting systemic inflammation or abnormal blood counts were observed (**Figure S1**). Ganglioside GM3 incorporated into CoPoP/PHAD/QS-21 liposome appeared safe as a vaccine formulation for mice.

To investigate the *in vivo* liposome uptake, the 2HPQ liposomes or the 2HPQ/GM3 liposomes were used. The QD655 signal intensity in the nearest draining lymph node to the injection site was recorded 24 h post-injection of indicated liposomes (**Figure 1E**). Both the 2HPQ (p = 0.011) and the 2HPQ/GM3 (p = 0.0008) liposomes showed an increased proportion of cells with liposome uptake, 10% and 15% separately, compared to the PBS-injected group (0%). The 2HPQ/GM3 group showed a higher average ratio of cells carrying liposomes with no significant difference from the 2HPQ group. The gating strategies are shown in **Figure S2A**. To visualize the loading of antigen to liposome and the uptake of liposome by CD169^+^ cells, a modified version of CP liposomes containing PoP lipids and GM3 (referred to as CPP/GM3) was loaded with HiLyte488-Renca peptides before incubating with freshly collected untreated splenocytes. As shown in **Figure S2B**, the CD169^+^ splenocyte is positive for liposomes and fluorescent peptides, while the surrounding CD169^-^ cells are negative for both liposomes and fluorescent peptides. **Figure 1F** shows a schematic diagram of different liposome formulations to better illustrate the liposomes used in this study. This schematic diagram does not represent the actual composition ratios or synthesis process. Liposome formulating components are shown with mass ratio in **Table S1**.

### Ganglioside-incorporated liposomes 2HPQ/GM3 target CD169^+^ cells

**Figure 2:**
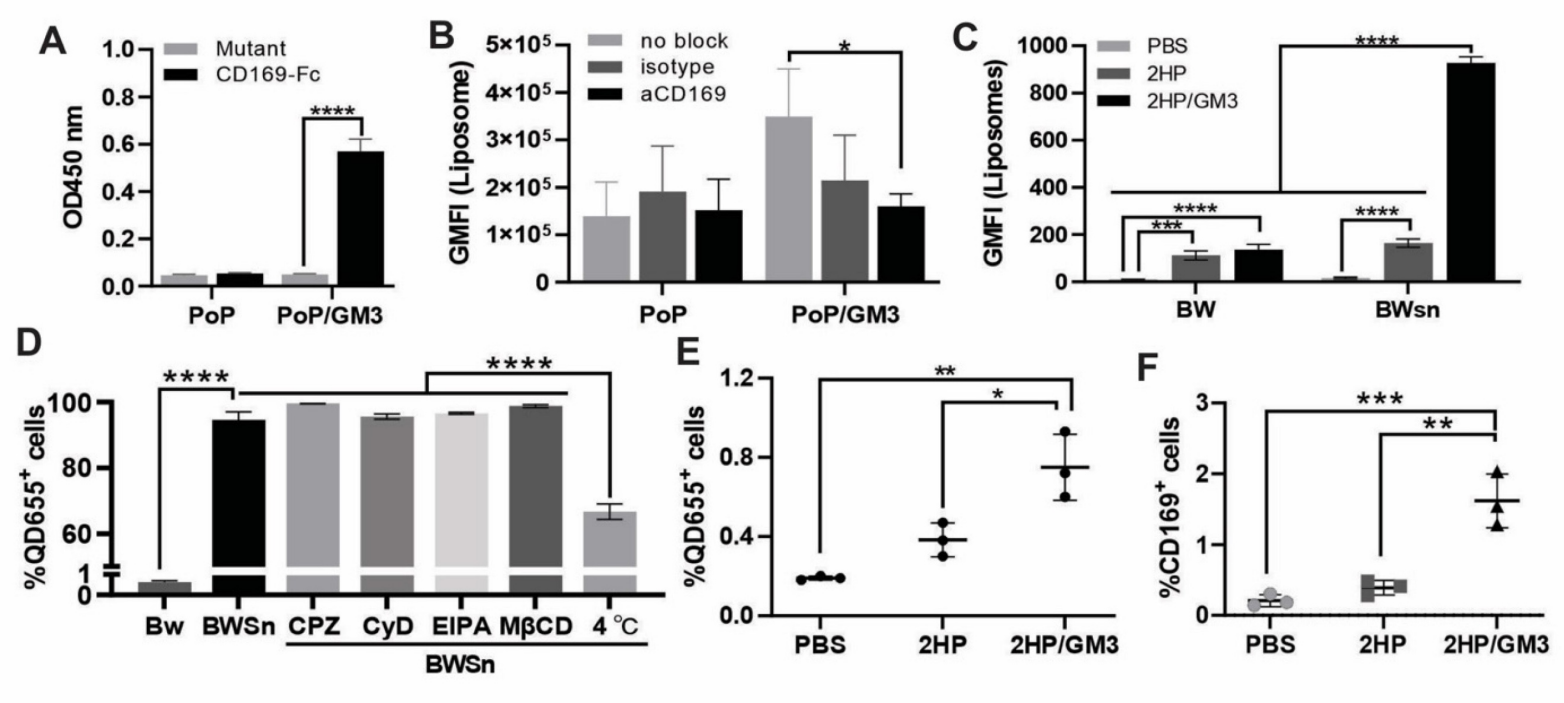
** CPQ-GM3 liposomes target CD169^+^ cells and carry short peptides. A)** Liposomes binding to wild-type or mutant mouse CD169 were determined in an ELISA-based assay (n = 3). **B)** Liposomes were added to moDC that were not blocked, or preincubated with a blocking aCD169 antibody or the isotype control. The liposome binding to moDC was quantified using flow cytometry analysis, indicated by the geometric mean fluorescence intensity (GMFI) (n = 5). Liposomes were incubated with BW cells or BWSn cells (expressing CD169). **C)** The uptake of liposomes by BW or BWsn cells was analyzed and quantified using flow cytometry analysis, indicated by the geometric mean QD655 signal intensity (n = 3). **D)** Percentage of QD655 signal-positive cells in BW or BWSn cells after 1 h incubation with 2HP/GM3 liposomes, and BWSn cells with or without preincubation at 4 °C or with endocytosis inhibitor (60 µM CPZ, 2 mM CyD, 100 µM EIPA or 10 mM MβCD) at 37 °C for 1 h (n = 3). **E)** Percentage of QD655 signal-positive cells and **F)** Frequency of CD169-expressing cells in all freshly collected living splenocytes after incubation with indicated liposomes for 1 h (n = 3). A) was analyzed by an unpaired t-test. Figures B-F were analyzed by One-way ANOVA followed by Tukey's multiple comparisons test. Error bars show mean ± std. dev. *, **, ***, and **** indicate *P* ≤ 0.05, 0.01, 0.001, and 0.0001, respectively.

The natural GM3 ganglioside used in this study is isolated from bovine milk and contains fatty acids varying in chain lengths and degree of bond saturation. To investigate whether the ganglioside-incorporated liposomes can target CD169^+^ cells, liposomes containing the cobalt-free fluorescent lipid porphyrin-phospholipid (PoP) were coated on an ELISA plate, and recombinant mouse CD169-Fc wild type (WT) or mutant (R97A) protein was added 25. The CD169-Fc wild-type protein bound to a greater extent than the mutant form when plates were coated with the natural ganglioside GM3 liposomes PoP/GM3 (p < 0.0001), indicating that binding to natural gangliosides is specific (**Figure 2A**).

In addition to testing the binding of ganglioside-containing liposomes to CD169 in an ELISA-based assay, liposome uptake studies were also performed with human CD169-expressing monocyte-derived dendritic cells (moDC). This functionalization with GM3 also resulted in CD169-specific targeting of PoP/GM3 liposomes to human moDC cells. The moDC cells were able to bind PoP/GM3 to the highest extent. When moDC were preincubated with CD169 blocking antibodies, a significant reduction in geometric mean fluorescence intensity (GMFI) (p = 0.0316) was detected for the PoP/GM3 liposome, while no significant differences were detected between the non-blocked condition and the isotype control antibody (**Figure 2B**). BW5147 cells (BW) and sialoadhesin-transduced BW5147 cells overexpressing CD169 (BWSn) were also used to assess uptake 26. In BWSn cells, higher levels of GM3 liposome uptake, represented by the QD655 signal geometric mean, were observed with statistical significance detected between the PoP/PHAD liposomes (referred to as 2HP liposomes) group and the 2HP liposome incorporated with GM3 (referred to as 2HP/GM3 liposomes) group (**Figure 2C**). Gating strategies and representative fluorescent microscope images are shown in **Figure S3A** and **Figure S3B**.

To understand the related endocytosis process, the BWSn cells were pretreated at 4 °C for 1 h to inhibit energy-dependent endocytosis or preincubated with clathrin-mediated endocytosis inhibitor Chlorpromazine hydrochloride (referred to as CPZ), actin polarization-mediated endocytosis inhibitor Cytochalasin D (referred to as CyD), micropinocytosis inhibitor EIPA, or cholesterol-mediated endocytosis inhibitor Methyl-β-cyclodextrin (referred to as MβCD). BW cells were incubated with 2HP/GM3 liposomes for 1h. Each inhibitor was freshly prepared at validated working concentrations (CPZ: 15-60 µM; MβCD: 1-10 mM; CyD: 2-10 µM; EIPA: 10-50 µM) and incubated with BWSn cells for 60 minutes before adding 2HP/GM3 liposomes. The percentage of 2HP/GM3 liposomes taken up by BW cells is under 1%, while BWSn cells show over 95% 2HP/GM3 uptake. None of the four classical endocytosis pathway inhibitors significantly decreased liposome uptake. The low-temperature condition, known to inhibit active endocytosis, showed significantly decreased liposome uptake from about 95% to 65% (**Figure 2D**). This indicated a CD169 expression-dependent and energy-dependent GM3-liposome internalization by BWSn cells.

The *in vitro* liposome uptake was visualized under a fluorescent microscope as shown in **Figure S4**. After incubation with 2HP/GM3 liposomes, the CD169-expressing cells BWSn exhibited the most abundant QD655 signal-positive population. The 2HP liposomes incubated BWSn cells, and 2HP/GM3 or 2HP liposomes incubated BW cells showed a low QD655 signal-positive cell population. This result further supports the statement that the uptake of GM3-incorporated liposomes by BWSn cells is mainly dependent on CD169 expression and the interaction between CD169 and GM3 molecules.

By incubating the indicated liposomes with splenocytes harvested from untreated mice *ex vivo* for 1 h, significantly increased GM3-incorporated liposome uptake was detected in the 2HP/GM3 group (0.75%) compared to the PoP/PHAD liposomes (2HP) group (0.4%) (**Figure 2E**). An increased detectable fraction of CD169-positive cells was observed in the 2HP/GM3 liposome group (1.6%) compared to the 2HP liposome group (0.3%), suggesting a preferential interaction between GM3-containing formulations and CD169⁺ antigen-presenting cells. This is consistent with the known ability of CD169 to recognize sialylated glycolipids such as GM3, which may lead to selective association or stabilization of CD169⁺ cells within the analyzed population (**Figure 2F**) 18, 27, 28. A total of 3x10^4^ cells per sample was collected for analysis, detailed representative gating strategies are shown in **Figure S3C**.

### Ganglioside-incorporated liposomes increase the antigen-specific CD8^+^ T cell population

**Figure 3:**
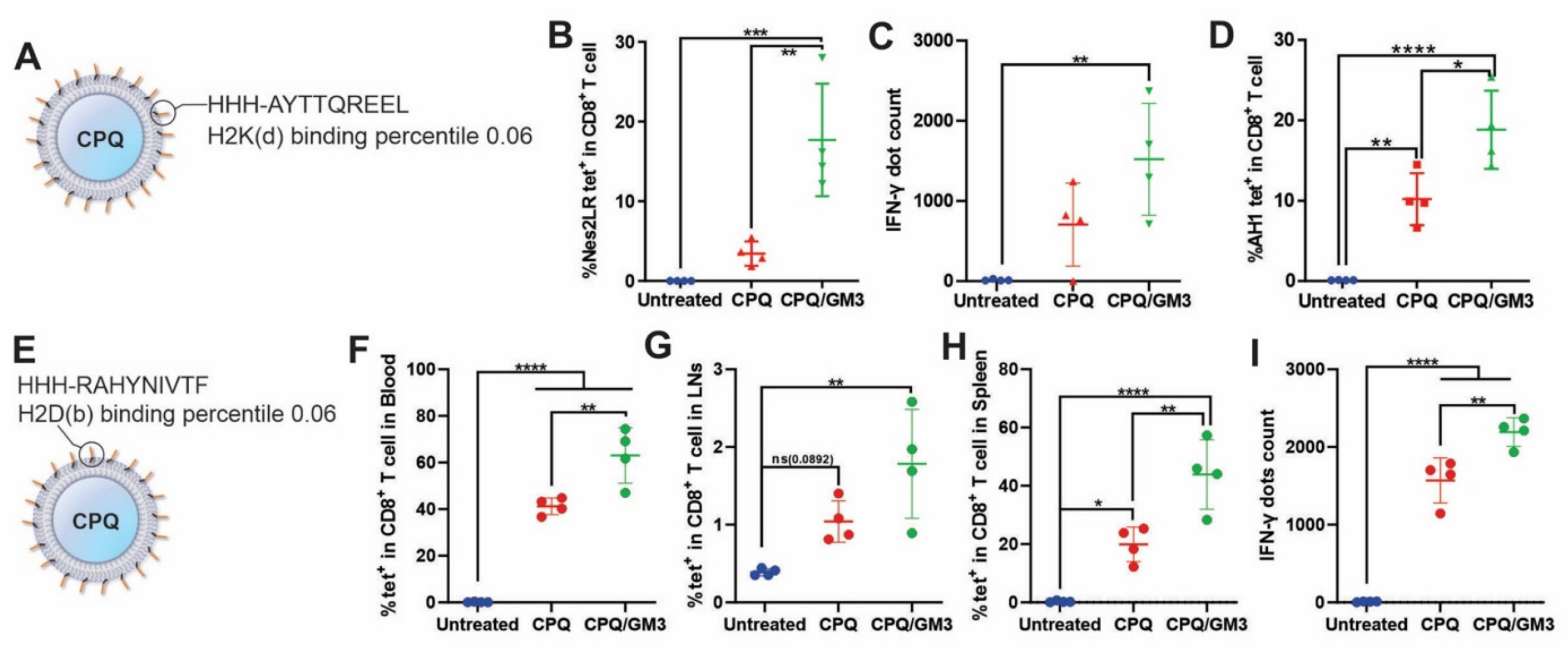
** Lipid peptide particles that include GM3 induce enhanced antigen-specific CD8^+^ T cell production.** Mice were vaccinated on day 0 and 7 with the indicated vaccines containing 2 μg peptides per mouse per injection. Blood, splenocytes, and lymph nodes were collected on day 14 for flow cytometry or IFN-γ ELISPOT analysis. **A)** Schematic of Nes2LR peptide bound onto CPQ liposome. **B)** Percentage of Nes2LR tetramer-specific CD8^+^ T cells in all CD8^+^ T cells in the blood. **C)** IFN-γ ELISPOT positive dot count using splenocytes.** D)** Frequency of AH1 tetramer-specific CD8^+^ T cells in all CD8^+^ T cells in blood. **E)** Schematic of E7 peptide bound onto CPQ liposome. Percentage of E7 tetramer-specific CD8^+^ T cells in all CD8^+^ T cells in **F)** blood, **G)** lymph node, or **H)** spleen. **I)** IFN-γ ELISPOT positive dot count using splenocytes. Data analyzed by one-way ANOVA with Tukey's multiple comparison test. Error bars show mean ± std. dev. *, **, ***, and **** indicate *P* ≤ 0.05, 0.01, 0.001, and 0.0001, respectively. Each data point represents an individual mouse.

To test the immunogenicity of ganglioside GM3-incorporated liposomes *in vivo*, short peptides antigen Nes2LR (**Figure 3A**), E7 (**Figure 3E**), and mimotope AH1-A5-bound liposomes were tested. These peptides are all strong binders to the MHC-I molecule predicted by the online MHC-I prediction tool NetMHC-4.0 with a percentile ranking of 0.06, 0.06, and 0.5, respectively 29. Mice received two peptide vaccine injections intramuscularly on days 0 and 7, separately. Blood, spleen, or lymph node samples were collected on day 14 for analysis. Antigen-specific CD8^+^ T cells were gated and analyzed by using flow cytometry. The murine IFN-γ ELISPOT kit was used to visualize and quantify IFN-γ secretion from splenocytes. Representative gating strategy, detailed IFN-γ ELISPOT well images and spots counting were shown in **Figure S5**. The use of CPQ/GM3 liposomes elevated the antigen-specific CD8^+^ T cell response for all three peptide vaccines compared to the CPQ liposome group. In blood samples for Nes2LR antigen, CPQ/GM3 liposomes induced 4 times higher antigen-specific CD8^+^ T cells population compared to CPQ liposomes (**Figure 3B**). Though no significant difference was detected in the splenocytes IFN-γ production, the CPQ/GM3 liposome group exhibited the increased average IFN-γ production in response to Nes2LR peptide stimulation (**Figure 3C**). The efficacy of GM3 varies for different antigens. According to previous studies, the wild-type peptide AH1 is not as immunogenic as the mimotope AH1-A5. With the addition of GM3 into CPQ liposomes, the AH1-specific CD8^+^ T cell population was further increased from an average of 10% to 18% (**Figure 3D**). Compared to the CPQ group, using the CPQ/GM3 liposomes showed a significant elevation of the E7-specific CD8^+^ T cell population in the blood (**Figure 3F**), lymph node (**Figure 3G**), and spleen samples (**Figure 3H**). No statistically significant difference detected between CPQ and CPQ/GM3 group in the lymph node sample. In response to the stimulation of E7 peptide, the CPQ/GM3 group showed increased production of splenocytes IFN-γ from average 1600 spots to average 2100 spots with significant difference (**Figure 3I**). Representative IFN-γ ELISPOT well images were shown in **Figure S5C**. Overall, the incorporation of the natural ganglioside GM3 into CPQ liposomes increased short peptide antigen immunogenicity.

### Ganglioside-incorporated liposomes improve therapeutic tumor-inhibiting efficacy

Having established the immunogenicity of peptide vaccines with ganglioside GM3, the next step is to verify whether vaccination with the natural ganglioside GM3 could improve anti-tumor activity. Based on our previous studies, when the first CPQ vaccination is given on days 8 for the therapeutic TC-1 tumor, and on days 6 for the therapeutic RENCA tumor, these large established tumors cannot be fully cured 13, 15. Here we compared CPQ or CPQ/GM3 delivered E7 or Nes2LR vaccine on suppressing TC-1 or RENCA tumor following the same treatment schedule to test whether CPQ/GM3 formulated short peptide vaccines could bring stronger tumor-inhibiting efficacy. Since the AH1-A5 loaded CPQ vaccine can fully eradicate the CT26 tumor in the mouse model, we are not testing the AH1-A5 loaded CPQ/GM3 vaccine in this study 14.

**Figure 4:**
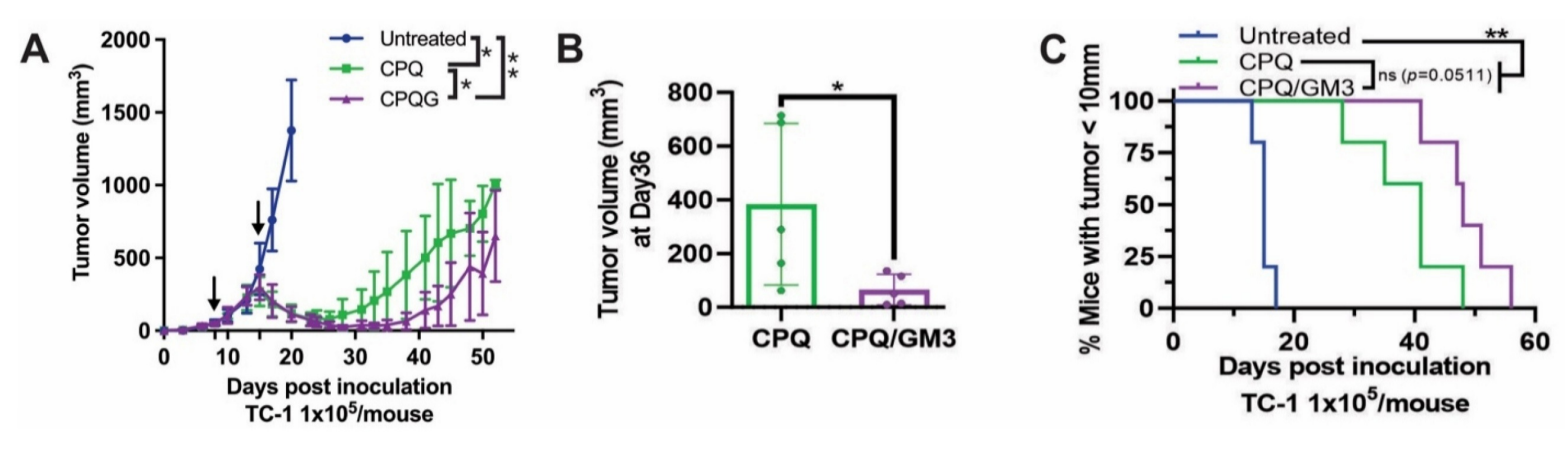
** CPQ/GM3 liposomal E7 peptide vaccine retarded the TC-1 tumor growth and prolonged the survival of mice.** Mice were inoculated with 1x10^5^ TC-1 tumor cells subcutaneously on day 0 and immunized with the indicated E7 vaccine, 2 µg per mouse per injection intramuscularly on day 8 and day 15. **A)** Average tumor volume. **B)** Tumor volume on day 36. **C)** Percentage of mice with tumor diameter under 10mm. Arrows indicate vaccination time points. Error bars show mean ± std. dev. for n = 4 per group. Figure 4A is analyzed by mixed-effects analysis followed by Tukey's multiple comparisons test. *, **, ***, and **** indicate *P* ≤ 0.05, 0.01, 0.001, and 0.0001, respectively. Figure 5B was analyzed by one-way ANOVA with Tukey's multiple comparisons test. Figure 5C was analyzed by Log-rank (Mantel-Cox) test.

As shown in **Figure 4A**, the first and second E7 peptide vaccinations were given on day 8 and day 15 after TC-1 tumor inoculation on day 0. The CPQ/GM3 group tumor volume decreased and relapsed on around day 35, while the CPQ group tumor relapsed on day 25. On day 36, the average tumor volume of the CPQ/GM3 group (50 mm^3^) was significantly smaller than the CPQ group (383 mm^3^) (**Figure 4B**). Mice from the CPQ/GM3 group required a longer time for tumor length to grow over 10 mm than mice from the CPQ group (P = 0.0511, **Figure 4C**). Both therapeutic tumor models verified the improved tumor-inhibiting efficacy of incorporating the GM3 into liposomal vaccines.

**Figure 5:**
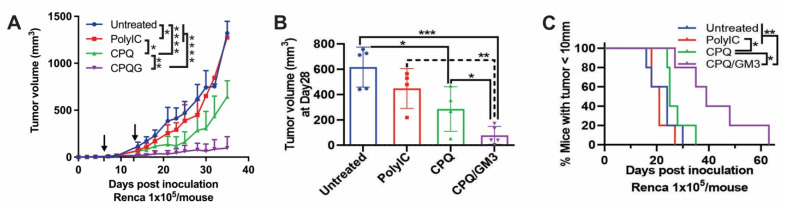
** CPQ/GM3 liposomal Nes2LR peptide vaccine retarded the Renca tumor growth and prolonged the survival of mice.** Mice were inoculated with 1x10^5^ Renca tumor cells subcutaneously on day 0 and immunized with the indicated 2 μg Nes2LR vaccine per mouse per injection intramuscularly on days 6 and 13. **A)** Average tumor volume. **B)** Tumor volume on day 28. **C)** Percentage of mice with tumor diameter under 10mm. Arrows indicate vaccination time points. Error bars show mean + or ± std. dev. for n = 4 per group. Figure 5A is analyzed by Mixed-effects analysis followed by Tukey's multiple comparisons test. *, **, ***, and **** indicate *P* ≤ 0.05, 0.01, 0.001, and 0.0001, respectively. Figure 4B, D, and E were analyzed by one-way ANOVA with Tukey's multiple comparisons test. Figure 4C was analyzed by the Log-rank (Mantel-Cox) test.

For comparison with liposomal vaccine formulations, Poly(I:C), a synthetic analog of double-strand RNA known as a TLR3 agonist, was used as a representative soluble immunostimulatory adjuvant 30. As shown in **Figure 5A**, when the first and second Nes2LR peptide vaccinations were given on day 6 and day 13 after Renca tumor inoculation on day 0, the CPQ/GM3 group exhibited lower average tumor volume compared with all other groups. On day 28, the CPQ/GM3 group average tumor volume (77 mm^3^) was lower than the Untreated (616 mm^3^), the PolyIC (448 mm^3^), and the CPQ groups (286 mm^3^) with significant difference (**Figure 5B**). Mice from the CPQ/GM3 group took over 60 days for all tumors to grow over 10 mm, while mice from the CPQ group took only 35 days. The difference was found to be significant (**Figure 5C**).

**Figure 6:**
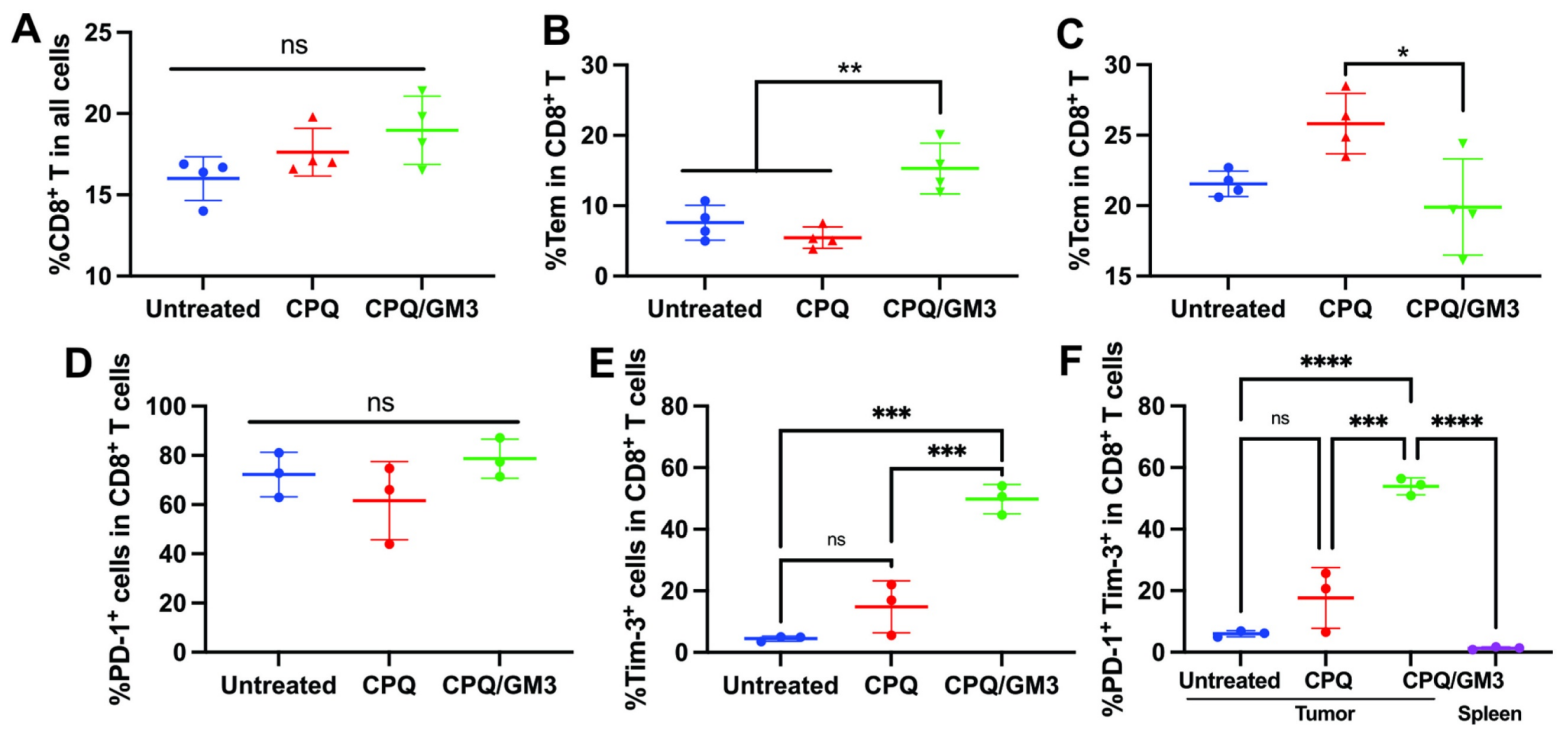
** CPQ/GM3 liposomal Nes2LR peptide vaccine affect CD8^+^ T cell differentiation and show high expression level of PD-1 and Tim-3. A)** percentage of CD8^+^ T cell population in all cells;** B)** percentage of Tem and **C)** Tcm population in all CD8^+^ T cell; The percentage of** D)** PD-1 expression level, **E)** Tim-3 expression level and** F)** PD-1 and Tim-3 co-expression in all CD8^+^ T cells. *, **, ***, and **** indicate *P* ≤ 0.05, 0.01, 0.001, and 0.0001, respectively. Figures were analyzed by one-way ANOVA with Tukey's multiple comparisons test.

To evaluate memory CD8^+^ T cell differentiation in Renca tumor-bearing mice, the effector memory and central memory subsets were analyzed in peripheral blood on day 20 following the same Renca tumor therapeutic challenge schedule. Compared to the untreated and the CPQ groups (5.37%), mice immunized with the CPQ/GM3 formulated vaccine exhibited no statistical difference on CD8^+^ T cell population (**Figure 6A**), but with a significantly increased CD8^+^ Tem cells (**Figure 6B**), decreased CD8^+^ Tcm cells (**Figure 6C**), and a corresponding shift in the memory phenotype distribution, as shown in the **Figure S6A**. This enrichment of Tem cells suggests a promoted effector-oriented CD8^+^ T cell differentiation, which is consistent with enhanced anti-tumor immune responses during ongoing tumor challenges.

The TC-1 and Renca tumor resistance was investigated in a previous study with high expression level of PD-1 on CD8^+^ T cells that infiltrating the tumor 31. We analyzed the PD-1 expression (**Figure 6D**), TIM-3 expression (**Figure 6E**), and co-expression of PD-1 and TIM-3 (**Figure 6F**) on these immunized Renca tumor bearing mice. High co-expression of PD-1 and TIM-3 was detected on tumor-infiltrating CD8^+^ T cells, but not in the CD8^+^ T cells from spleen (**Figure 6F**). Gating strategy and representative PD-1 expression, Tim-3 expression and co-expression of these two signals were shown in **Figure S6B**. PD-1 and Tim-3 co-expression indicates the CD8^+^ T cell exhaustion, which was reported to inhibit how the cytotoxic T cells perform tumor cell clearance.

## Discussion

We assessed the potential of incorporating the natural ganglioside GM3 lipid into CoPoP/PHAD/QS-21 (CPQ) liposomes to enhance the immunogenicity of short MHC class I epitopes. We have previously shown that the His-tagged antigen-capturing lipid CoPoP and the immunostimulatory adjuvants QS-21 are required for the CPQ delivery system to initiate anticancer immune responses. Incorporating GM3 into CPQ liposomes brings no significant changes in liposome size and the ability for peptide antigen binding. The CPQ/GM3 liposomal nanoparticles remained below 200 nm in diameter, which is a preferred size for efficient lymphatic drainage and lymph node targeting. In a GM3 dosage study, GM3 concentration range was used from 0 to 5 mol% for targeting human and murine cells 28. The usage of GM3 in the present study was about 2 mol%. Thus, this dosage is within the previously reported range of GM3 concentration for providing targeting. However, a more comprehensive GM3 dose study with our CPQ liposome will further benefit the CPQ/GM3 liposomal platform development.

Following a single intramuscular injection, liposomes containing GM3 were more readily taken up by CD169-expressing macrophages within the lymph nodes compared to GM3-free formulations. This indicates that GM3 enhances the trafficking of CPQ liposomes through the lymphatic system while preserving their capacity for targeted immune cell engagement.

*In vitro* and *ex vivo* assays revealed that decorating liposomes with GM3 increased their uptake by CD169-expressing cells. CD169 is known to serve as an adhesion and endocytic receptor for sialic acid-containing glycans, such as the trisaccharide GM3 used in our liposome formulation 32. CD169-negative BW cells were significantly less efficient at internalizing GM3-containing liposomes compared to CD169-expressing BWSn cells. Furthermore, the uptake of GM3-incorporated liposomes by BWSn cells did not appear affected by inhibitors of actin polymerization, cholesterol depletion, clathrin-dependent endocytosis, or micropinocytosis. The decreased uptake observed at 4 °C suggests an energy-dependent mechanism, consistent with previous reports that CD169 mediates internalization of GM3-functionalized particles through non-classical pathways, even in non-phagocytic cells 27, 33. Such trafficking routes may impact antigen retention and intracellular routing favorable for antigen presentation. Further mechanistic studies will be required to precisely define the internalization route.

In the *ex vivo* assay, untreated splenocytes incubated with GM3-incorporated liposomes exhibited an increased liposome uptake and a higher population of CD169-expressing cells compared to liposomes without GM3. A similar trend of increased CD169^+^ cell populations in lymph nodes was observed 24 h post-single intramuscular vaccination, although differences were not statistically significant. Collecting lymph nodes at various time points post-vaccination may provide better insights into cell preferences and liposome retention kinetics. Further investigation about whether GM3-incorporated liposomes induce expansion or recruitment of CD169^+^ expressing cells is needed. To represent formulation-dependent differences within each experimental system, the GMFI and percentage-based analysis were applied to interpret the uptake of liposome *in vitro* or *ex vivo* separately.

While GM3-incorporated liposomes demonstrated enhanced uptake by CD169-expressing cells, the direct visualization of liposome binding to CD169⁺ cells within spleen and lymph node samples by flow cytometry remains technically challenging due to the low abundance of this population in secondary lymphoid tissues. In our analysis, CD169⁺ cells typically represented less than 1% of total splenocytes, which limits further sub-gating. Complementary *in vitro* studies performed using CD169-expressing cell lines allows direct assessment of liposome-CD169 interactions and provided clear evidence supporting GM3-mediated targeting. Together with the *ex vivo* uptake observed in splenocyte and lymph node samples, these data support a model in which GM3 incorporation enhances preferential interaction with CD169⁺ antigen-presenting cells. Future studies aimed at enriching or isolating CD169⁺ populations will further elucidating the intracellular trafficking and antigen processing pathways of GM3-incorporated liposomes within these cells.

We also evaluated the safety profile of GM3 and found no sign of autoimmune responses against this naturally occurring molecule in mice receiving our liposomal formulation. The antigen-loading efficiency of CPQ/GM3 liposomes was confirmed via quenching assays. The uptake of CPQ/GM3 liposomes by CD169-expressing cells was demonstrated through *ex vivo* and *in vivo* studies using splenocytes and BWSn cells, respectively. According to the Fluorescent Resonance Energy Transfer between the HiLyte488 and CoPoP lipid, the cobalt ion in CoPoP lipid quenches the emission light of HiLyte488 and PoP lipid. We have taken a set of images showing only CD169^+^ splenocytes showed positive signals for both CPQ/GM3 liposomes and HiLyte488-Nes2LR, while surrounding CD169-negative cells exhibited no evidence of antigen or liposome uptake. However, due to the low abundance of CD169^+^ splenocytes and the challenges of culturing and expanding these cells *in vitro*, we were unable to directly visualize uptake across multiple liposome formulations in this cell type.

Incorporation of natural ganglioside GM3 into CPQ liposomes significantly enhanced the immunogenicity of short MHC class I epitopes, including E7_49-57_, enhanced mimotope AH1-A5, and the neoantigen Nes2LR. The mechanisms likely involve improved targeting of liposomes to CD169^+^ macrophage cells, enhancing antigen uptake, as mentioned above and demonstrated in previous studies 17, 18. Two therapeutic murine tumor models are used in this study with different vaccination schedules to approach the limit of CPQ/GM3 vaccine efficacy, where the CPQ liposomal vaccine fails to inhibit established tumors. In both therapeutic Renca and TC-1 tumor challenges, CPQ/GM3 group retarded tumor growth and prolonged mice survival with a significant difference from the CPQ group. In the TC-1 therapeutic challenge, the CPQ/GM3 group tumor average volume decreased from 300 mm^3^ (day 15) to 25 mm^3^ (day 30) after two vaccinations on day 8 and 15 separately. These findings suggest that GM3 incorporation enhances the efficacy of the liposome vaccine formulation, making it a promising candidate for cancer peptide vaccine development. The Poly(I:C) delivered Nes2LR peptide vaccine was limited in inducing tumor-inhibiting activities. This may reflect its role as an immune stimulant, rather than a delivery system that improves antigen presentation. This is addressed in a recently published paper by co-delivery Poly(I:C) and short peptide antigen in lipid nanoparticle 34.

In this study, mainly two antigenically distinct short peptide antigens were applied, Nes2LR and E7. They were formulated in liposomal vaccine platforms and contributed to differences in baseline immune responses. Notably, in both antigen models, incorporation of GM3 into the liposomal formulation enhanced antigen-specific immune responses compared with the corresponding GM3-free formulations. These findings indicate that while antigen-specific factors determine the baseline magnitude of immune activation, GM3 incorporation provides an additional enhancement, supporting the utility of GM3-decorated liposomes as a general immune-targeting strategy.

While promising, these antigens loaded CPQ/GM3 vaccines were unable to fully eradicate large established tumors, and relapse was observed following initial tumor regression. With further investigation of Renca tumor microenvironment, we detected co-expression of PD-1 and TIM-3 on tumor-infiltrating CD8^+^ T cells. This indicates a signal of exhausted CD8^+^ T cell that unable to further kill tumor cells. These relapse models provide a valuable platform for future investigations into the tumor microenvironment (TME), including the role of immune checkpoint molecules 35. The combined use of the peptide liposomal vaccine with immune checkpoint blockade could overcome this challenge. Additional studies on the vaccine dose response and the addition of other possible adjuvants may bring more promising results for tumor inhibition and the growth reversal of even larger tumors.

Several limitations of this study should be noted. The impact of GM3 concentration and lipid density was not systematically assessed. Future work will also focus on identifying GM3 functional compounds and spending more effort on assessing and optimizing lipid side-chain lengths to further enhance immunogenicity and advance vaccine development. Furthermore, anti-tumor impact of GM3 targeting was in murine models, which may not be fully representative of other species. Future studies should be planned to better examine the translational potential of this approach.

## Conclusion

Ganglioside GM3 incorporated CoPoP/PHAD/QS-21 liposomes can capture antigen peptides to form nanoparticles and target CD169^+^ cells to increase antigen immunogenicity and inhibit tumor growth in mouse tumor models. The CPQ/GM3 liposome appears to be a promising antigen delivery tool for cancer peptide vaccine development.

## Materials and Methods

### Liposome preparation

The 2HPQ, CPQ, CPP/GM3, 2HP/GM3, and CPQ/GM3 liposomes were produced by ethanol injection and nitrogen-pressurized lipid extrusion methods 24, 36. PBS and pure ethanol were prewarmed to 60°C, lipids were weighted at the ratio of DOPC:Chol:Copop:PHAD (20:5:1:0.4), DOPC:Chol:Copop:GM3:PHAD (20:5:1:1:0.4), and DOPC:Chol:PoP:PHAD (20:5:1:0.4). Lipids were dissolved in 1 mL ethanol for 10 min, followed by sonication and diluted with 4 mL PBS for 10 min at 60 °C. Incubated liposomes were filtered through a 0.45 μm filter before extruding through the stack of 200,100, and 80 nm membrane filters. The nitrogen-pressurized liposome extruder (Northern lipids) was used at 55 °C and dialyzed in PBS at 4 °C overnight to remove excess ethanol and stored at 4 °C. Two μL liposomes were added to 1 mL PBS for size and polydispersity index measurement using dynamic light scattering. Concentration of liposomes was determined by the standard curve of pure CoPoP or PoP at absorbance 645 nm. QS-21 was added into the synthesized liposome at the ratio of CoPoP: QS-21 (1:0.4) and incubate at 4 °C overnight. For every 50 µL CPQ or CPQ/GM3 vaccine per mouse per injection, the concentration of each liposomal component is 4000 µg/mL DOPC, 800 µg/mL Cholesterol, 160 µg/mL CoPoP, 64 µg/mL PHAD, 64 µg/mL QS-21 with or without 160 µg/mL ganglioside GM3 for indicated formulation. Lipids used for liposomes production were 1,2-dipalmitoyl-sn-glycero-3-phosphocholine (DOPC, Corden # LP-R4-076, cholesterol (PhytoChol, Wilshire Technologies), synthetic Monophosphoryl Hexa-acyl Lipid A, 3-Deacyl (PHAD-3D6A, Avanti Cat # 699855), ganglioside GM3 (Bovine Milk) (Avanti, Cat # 860058). Liposome formulations used in this study are further detailed in the Table S1.

### Cryo-EM microscopy

A volume of 25 μL CPQ, CPQ/GM3 liposomes was added to 175 μL of PBS buffer. Sample vitrification was done with a Vitrobot Mark IV (Thermo Fisher Scientific). Holey carbon grids (C-Flat 2/2-3Cu-T) were washed with chloroform for 2 h before sample vitrification. Grids were subjected to negative glow discharge in air at 5 mA for 15 seconds before the sample application. 3.6 μL of liposomes were applied to the holey carbon grids and blotted with Vitrobot blotting paper (Standard Vitrobot Filter Paper, Ø55/20 mm, Grade 595). 3.6 μL of the same liposome sample was then applied a second time to the same holey carbon grid, and the grid was blotted once in the Vitrobot for 3 sec using a blot force of +1 prior to plunging into liquid ethane. The Vitrobot temperature was set to 25°C with 100% relative humidity.

Data acquisition was performed using SerialEM software on a Titan Krios electron microscope acquired at 300kV 37. Images collection used a Gatan K3 direct electron detector equipped with a Bioquantum imaging filter. Nominal defocus was set to -2.00 µm. Images were collected using a total exposure of 50 e-/Å2 at a nominal magnification of 81,000× corresponding to a calibrated pixel size of 1.09 Å.

### Vaccine preparation

All peptides were synthesized by Genscript with 3 His-tag added to the N-terminus of all peptides: Nes2LR (AYTTQREEL), E7 (RAHYNIVTF), and AH1 enhanced mimotope AH1-A5 (SPSYAYHQF) referred to as AH1. Peptide lyophilized powder was reconstituted by sterile Di-Water or PBS to 1 mg/mL stock solution as recommended by Genscript. Peptide and liposome were co-incubated with the mass ratio of CoPoP: peptide at 4:1. 2 μg peptide from 80 μg/mL stocking solution was admixed with 8 μg CoPoP lipid-containing CPQ or CPQ/GM3 liposome from 320 μg/mL stocking solution at room temperature for 1 h. To prepare for injection in mice, the incubated samples were mixed with PBS and adjusted to a final volume of 50 μL per injection. Poly(I:C) vaccine was prepared by admixing 2 μg Nes2LR short peptide antigen with 100 μg Poly(I:C) (InvivoGen) per mouse per vaccination before use. Each injection contained 2 µg of antigen for one mouse.

### Fluorescence measurements

His-tagged fluorescent Nes2LR peptide and His-tagged Nes2LR peptide were mixed at a 1:9 mass ratio (80 μg/mL, 2 μg) before they were incubated with PBS, 2HPQ, CPQ, or CPQ/GM3 liposomes (320 μg/mL, 8 μg) separately for 2 h at room temperature. Samples were wrapped with aluminum foil paper and avoided from direct light. After mixing 40 μL prepared samples with 3 mL PBS, the emission wavelength intensity of samples was recorded from 500 nm to 600 nm with excitation wavelength at 480 nm with 1-second integration by Photon Technology International PTI QuantaMaster Fluorescence/Luminescence Spectrometer.

For the FRET binding test, His-tagged fluorescent Nes2LR peptide and His-tagged Nes2LR peptide were mixed at a 1:9 mass ratio (80 μg/ml, 2 μg). Mixed His-tagged Nes2LR was incubated with 2HPQ, CPQ, CPQ/GM3 liposomes (320 μg/mL, 8 μg) separately. Mixed samples were collected after different incubation times. Dilute 4 uL incubated sample with 196 uL PBS, triply prepared samples were read by NanoBrook 90Plus PALS instrument with excitation/emission 497 nm/526 nm. The FRET binding percentage was calculated according to the Hilyte488 signal loss. Percent of binding = (1-(Hilyte488 intensity in test samples / Hilyte488 intensity in PBS)) 100%.

### CD169 Fc ELISA

Liposomes were 100× diluted in ethanol to a final concentration of 1.6 µg/mL on Immuno MaxiSorp plates (NUNC, Roskilde, Denmark). Coated plates were blocked with 1% BSA in PBS (BSA; Fraction V, Fatty acid free, Calbiochem, San Diego, CA, USA). Samples were then incubated with wildtype or mutant (R97A) CD169 Fc (2 μg/mL) for 1 h at room temperature. Peroxidase-conjugated goat anti-human IgG (Jackson ImmunoResearch, Ely, UK) was added for an additional 30 minutes after which the plates were washed. As a substrate, TMB (Sigma Aldrich, Darmstadt, Germany) was added and the optical density (OD) was measured in a microplate absorbance spectrophotometer (Biorad, Hercules, CA, USA) at 450 nm.

### Liposome binding to monocyte-derived dendritic cell (moDC)

Human peripheral blood mononuclear cells were isolated from buffy coats (Sanquin, Amsterdam, the Netherlands) 38. In short, PBS containing 1% citrate was used to mix the blood and the mixture was carefully layered on top of Lymphoprep (Alere Technologies AS, Oslo, Norway) and centrifuged for 30 minutes at 800g. Cells were collected and added to a Percoll (GE Healthcare, Chicago, USA) layer and centrifuged for 10 minutes at 300g. The monocyte layer was isolated and washed three times before they were resuspended in RPMI supplemented with 10% FCS (Biowest, Manassas, VA, USA), 50 U/mL penicillin (Lonza, Basel, Switzerland), 50 μg/mL streptomycin (Lonza, Basel, Switzerland) and 500 μg/mL IL-4 (ImmunoTools, Friesoythe, Germany) and 800 μg/mL Granulocyte Macrophage Colony stimulating Factor (GM-CSF) (ImmunoTools, Friesoythe, Germany) and cultured for 6 days to generate moDC. On day 4, moDC were pretreated with type I interferon (IFN) (100 IU/mL) to upregulate the expression of CD169. For liposome uptake experiments, moDC were plated, and 1.6 µg/mL liposomes were added. Where indicated, a CD169-blocking antibody (10 μg/ml clone 7.239) or an isotype control anti-Langerin (10 μg/ml clone 10E2 (produced in house)) was added to the moDC 15 min prior to liposome incubation. After 45 minutes, the cells were washed and stained with a Fixable Viability Dye eFluor 780 (eBioscience, San Diego, CA, USA). Subsequently, cells were fixed in 2% PFA (Electron Microscopy Sciences, Hatfield, PA, USA) and resuspended in 0.5% BSA in PBS until flow cytometry analysis.

### BW and BWsn uptake liposome and confocal imaging

BW5147 (BW) and BW5147Sn (BWsn) cells were cultured in RPMI supplemented with 10% FBS, 1% penicillin, and 1% streptomycin 26. In a 96-well U-bottom plate, 10 µg/mL CoPoP-containing liposomes were added to 3x10^5^ BW or BWsn cells. After 90 minutes of incubation at 37 °C with 5% CO_2_, cells were washed once with PBS and resuspended in 0.5% BSA in PBS for flow cytometry analysis.

For fluorescent confocal microscope imaging, 3x10^5^ BW or BWsn cells were incubated with 2HP or 2HP/GM3 liposomes containing 0.4 μg PoP lipids. After 1 h of incubation at 37 °C with 5% CO_2_, cells were washed with PBS and fixed with 4% Paraformaldehyde in PBS (Thermo Scientific, catalog no. J61899.AK). Cells were then washed with PBS and dried on slides. For each smear, 10 μL of Antifade Mounting Medium with DAPI (VECTASHIELD, catalog no. H-1200) was applied before applying the cover slides and sealing with nail polish. Prepared slides are stored at 4 °C and are kept away from direct light before imaging.

### Endocytosis inhibition

In the 96-well U-bottom plate, 5x10^5^ BW or BWsn cells in complete culture medium were preincubated with the following endocytosis inhibitor at indicated final inhibitor concentration for 1 h at 37 °C with 5% CO_2_: 1 mM, 5 mM, or 10 mM Methyl-β-cyclodextrin (MβCD, MedChemExpress, catalog no. HY-101461); 2 µm, 5 µm, or 10 µm Cytochalasin D (CyD, MedChemExpress, catalog no. HY-N6682); 10 µm, 25 µm, 50 µm, or 100 µm EIPA (MedChemExpress, catalog no. HY-101840); 15 µm, 30 µm, or 60 µm Chlorpromazine hydrochloride (CPZ, MedChemExpress, catalog no. HY-B0407A). Or cells were sitting in a 4 °C fridge for 1 h. For each well, 2.67 µg/mL PoP/PHAD/GM3 liposomes (2HPG) were added and incubated with cells for 1 h, followed by 2 times PBS wash. Cells were visualized under the fluorescent microscope and were ready for flow cytometry analysis. The percentage of QD655 signal-positive cells indicates the percentage of cells carrying liposomes formed with PoP lipid.

### Murine and cell studies

Balb/c mice (Charles River, 6-7 weeks old, female) and C57bl/6 mice (Jackson Laboratory, 6-7 weeks old, female) were randomly housed separately in a maximum of 4 mice per cage and maintained at the Comparative Medicine and Laboratory Animal Facilities (CM-LAF) of the University at Buffalo, State University of New York (IACUC Protocol# BME13028Y).

Mice received 2 intramuscular vaccinations at 7-day intervals, followed by blood and splenocytes collection 7 days after the second vaccination. Mice received a single vaccination of 10 µg 2HPQ or 2HPQG liposomes, followed by lymph nodes near the injection site were harvested 24 h post-injection.

Renca tumor cells and TC-1 tumor cells were purchased from the ATCC and cultured in Dulbecco's modified Eagle's medium and RPMI medium separately with 10% fetal bovine serum (FBS) and 1% 100 U/mL penicillin and 100 μg/mL streptomycin. Cells were cultured at 37 °C with 5% CO_2_. For the therapeutic challenge, mice were anesthetized with isoflurane and inoculated with 1x10^5^ Renca or TC-1 cells subcutaneously in the left flank. The tumor length and width were measured by caliper and volume was calculated with the formula: “Length × Width^2^ × 3.14 / 6”.

### Flow cytometry

Cells from the lymph node were separated before being stained with APC-labeled anti-mouse CD169 (Siglec-1) Antibody (Biolegend, Catalog: 142417, 200× final dilution) and aqua fluorescent reactive dye (Invitrogen, catalog no. L34957, 500× final dilution) for 30 min with constant shaking at 4 °C.

Fresh blood was collected in heparin-containing blood collection tubes. 60 µL of blood was collected in a tube with lithium heparin, followed by incubation with PE-labeled tetramer (NIH tetramer core facility, 500× final dilution) at 4 °C for 30 minutes with shaking. A mixture of Aqua fluorescent reactive dye labeled Live/Dead staining (500× final dilution), APC-CD8 antibody (200× final dilution), FITC-CD4 antibody (200× final dilution), and purified anti-mouse CD16/32 antibody (100× final dilution) was added to the sample and incubated for 1 h at 4 °C with shaking. Cells were then processed with RBC lysis buffer, washed with DPBS, and resuspended with 200 μL FACS buffer for flow cytometry using BD LSRFortessa X-20 cytometer. Data were further processed by FlowJo (version 10.6.2).

### ELISPOT

Spleens were harvested 7 days after the second vaccination. ELIPOST assay was performed using a Mouse IFN-γ Single-Color ELISPOT kit (ImmunoSpot). 3x10^5^ splenocytes in 100 μL 37°C warmed CTL-Test Medium (included in Kit) were added to each well, followed by the addition of 100 μL 20 μg/mL antigen peptide in CTL-Test Medium. Splenocytes were cultured for 24 h at 37°C with 5% CO_2_. IFN-γ capture, detection, and development steps were carried out as per manufacturer instructions.

### Statistical analysis

Data were analyzed by Unpaired t test, One-way ANOVA followed by Tukey's multiple comparisons test, or Log-rank (Mantel-Cox) tests by Prism 9 as indicated in figure captions. *, **, ***, and **** indicate p < 0.05, 0.01, 0.001, and 0.0001, respectively. All values were reported as ± standard deviation (S.D.) or Standard Error of Mean (SEM).

## Supplementary Material

Supplementary figures and table.

## Data Availability

All raw data are available from the authors upon reasonable request.
