# Supplementary material for: Ganglioside enhances the immunogenicity of nanoparticles displaying short synthetic tumor neoepitopes and epitopes
Source: Theranostics. 2026 Mar 17;16(10):5393–405. doi: 10.7150/thno.128187 (PMC13080682; doi:10.7150/thno.128187)
Supplement: Supplementary file 1 — Supplementary figures and table. [file thnov16p5393s1.pdf]

Supporting Information: Ganglioside enhances the immunogenicity of nanoparticles displaying short synthetic tumor neoepitopes and epitopes

Shiqi Zhou[1], Yuan Luo[1], Maarten K Nijen Twilhaar[2], Wei-Chiao Huang[1], Amal Seffouh[3], Yiting Song[1], Breandan Quinn[1], Sriram Neelamegham[5], Joaquin Ortega[3], Joke M.M. den Haan[2], Jonathan F Lovell[1]*

Table S1. Liposome types and formulating components shown with mass ratio.

<table>
<tr><th>Liposome</th><th colspan="7">Component mass ratio ("/"means not included)</th></tr>
<tr><td></td><td>DOPC</td><td>Cholesterol</td><td>CoPoP</td><td>PoP</td><td>PHAD</td><td>QS-21</td><td>GM3</td></tr>
<tr><td>PoP</td><td colspan="2" rowspan="10">20:5</td><td>/</td><td rowspan="6">1</td><td>/</td><td>/</td><td>/</td></tr>
<tr><td>PoP/GM3</td><td>/</td><td>/</td><td>/</td><td>1</td></tr>
<tr><td>2HP</td><td>/</td><td rowspan="8">0.4</td><td>/</td><td>/</td></tr>
<tr><td>2HP/GM3</td><td>/</td><td>/</td><td>1</td></tr>
<tr><td>2HPQ</td><td>/</td><td rowspan="4">0.4</td><td>/</td></tr>
<tr><td>2HPQ/GM3</td><td>/</td><td>1</td></tr>
<tr><td>CPQ</td><td rowspan="3">1</td><td>/</td><td>/</td></tr>
<tr><td>CPQ/GM3</td><td>/</td><td>1</td></tr>
<tr><td>CP</td><td>/</td><td>/</td><td>/</td></tr>
<tr><td>CPP/GM3</td><td>0.6</td><td>0.4</td><td>/</td><td>1</td></tr>
</table>

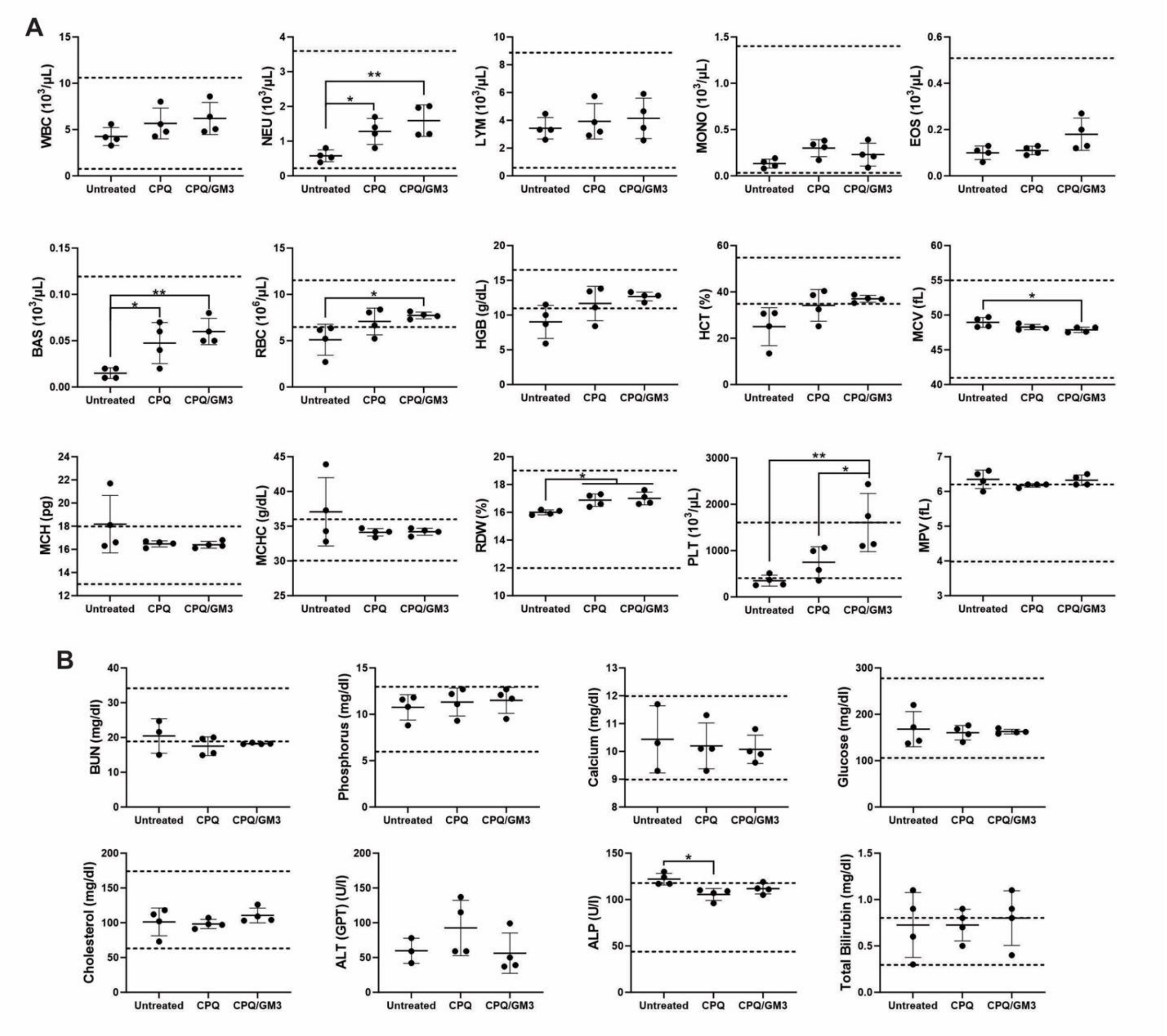

Figure S1. Safety profile of CPQ or CPQ/GM3 vaccines in immunized mice. A) CBC and **B)** Serum panel of mice received a single intramuscular injection of the indicated vaccine (n = 3). Figures were analyzed by One-way ANOVA followed by Tukey's multiple comparisons test. Error bars show mean ± std. dev. *, **, ***, and **** indicate $P \leqslant 0.05$, 0.01, 0.001, and 0.0001, respectively.

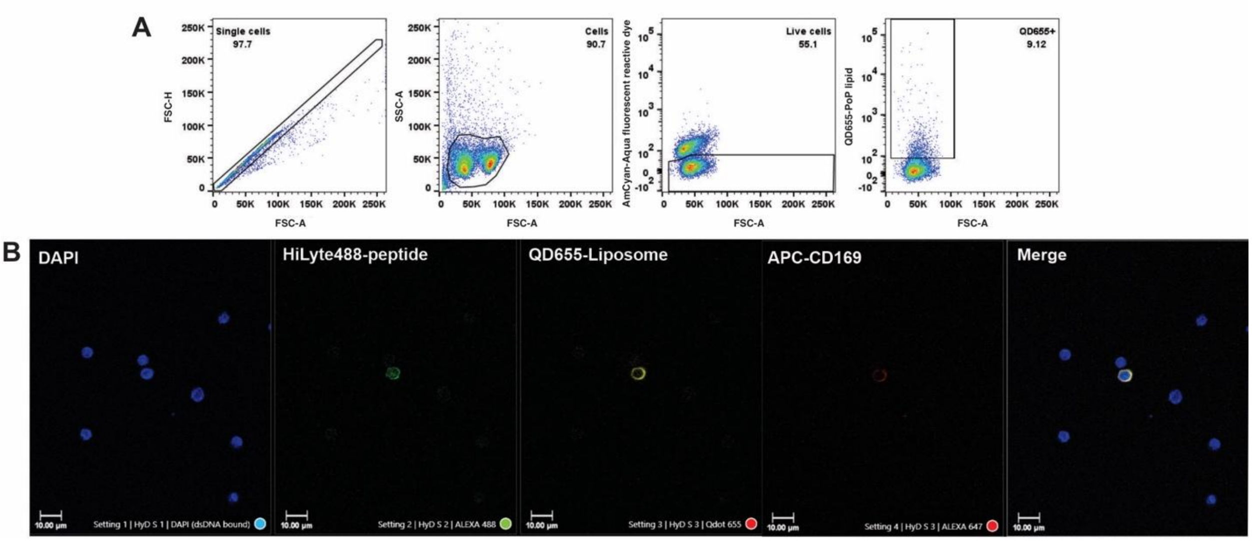

Figure S2. Representative flow cytometry gating strategies, fluorescent images. A) Representative gating strategies targeting CD169-expressing cells in the nearest draining lymph node 24 h post-injection of 2HPQ/GM3 liposomes. **B)** CD169^{+} (red) splenocyte (blue) is positive for liposomes (yellow) and fluorescent peptides (green), while the surrounding CD169^{+} cells are negative for both liposomes and fluorescent peptides.

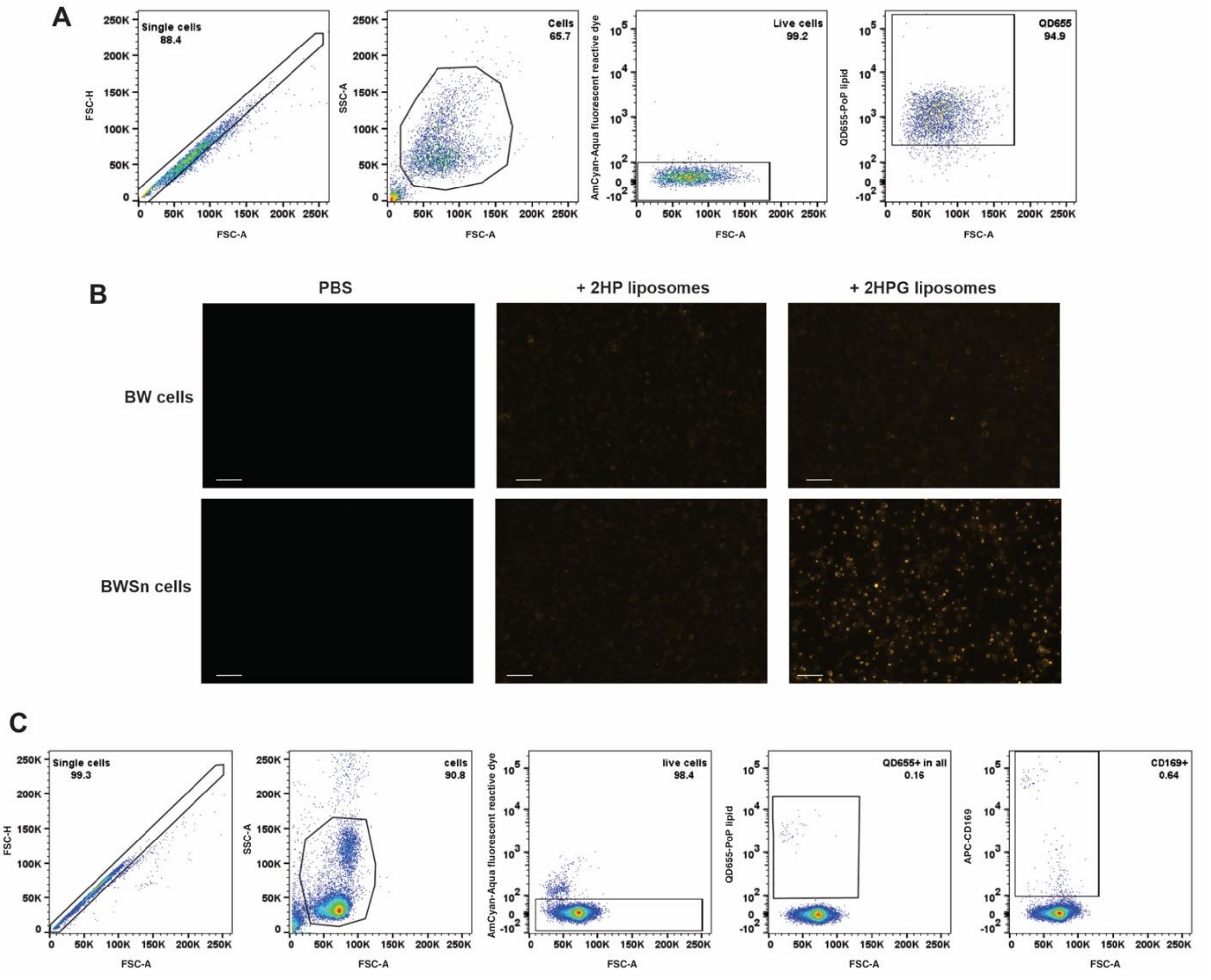

Figure S3. Representative flow cytometry gating strategies and fluorescent images. A) Representative gating strategies and **B)** representative fluorescent microscope images for targeting QD655 signal-positive cells after incubating BW or BWsn cells with 2HP liposomes or 2HP/GM3 liposomes. Scale bar indicates 150 µm. **C)** Representative gating strategies for targeting CD169-expressing cells or QD655-positive cells in all freshly collected living splenocytes after incubation with indicated liposomes for 1 h.

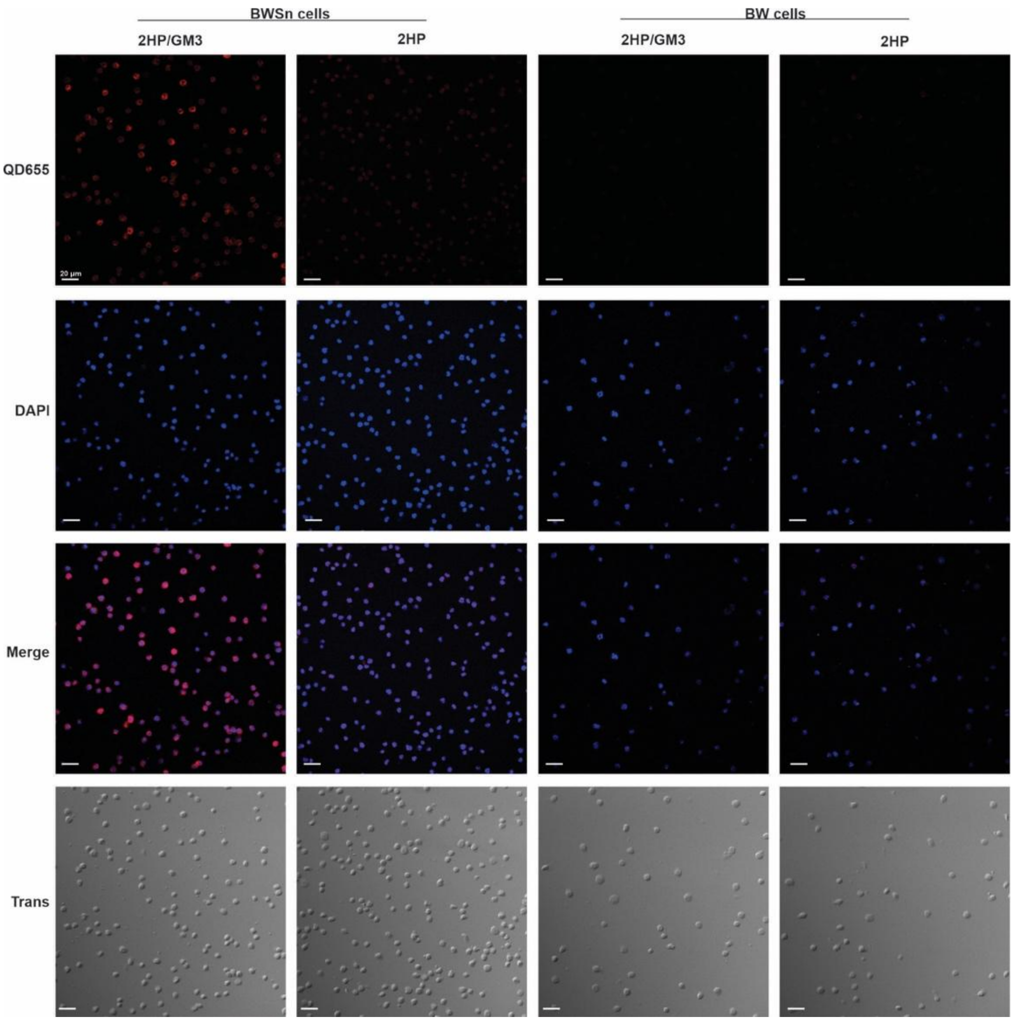

Figure S4. 2HP or 2HP/GM3 liposome taken up by BWSn or BW cells visualized under a fluorescent confocal microscope. 3x10^5 BW or BWSn cells were incubated with 0.4 μg 2HP or 2HP/GM3 liposomes at 37 °C for 1 h. Cells were fixed using 4% PFA and dried on slides. Smear was stained with 10 μL mounting buffer with DAPI, sealed with a cover slide, and nail polish. Slides were stored at 4 °C before imaging. Scale bar indicates 20 μm.

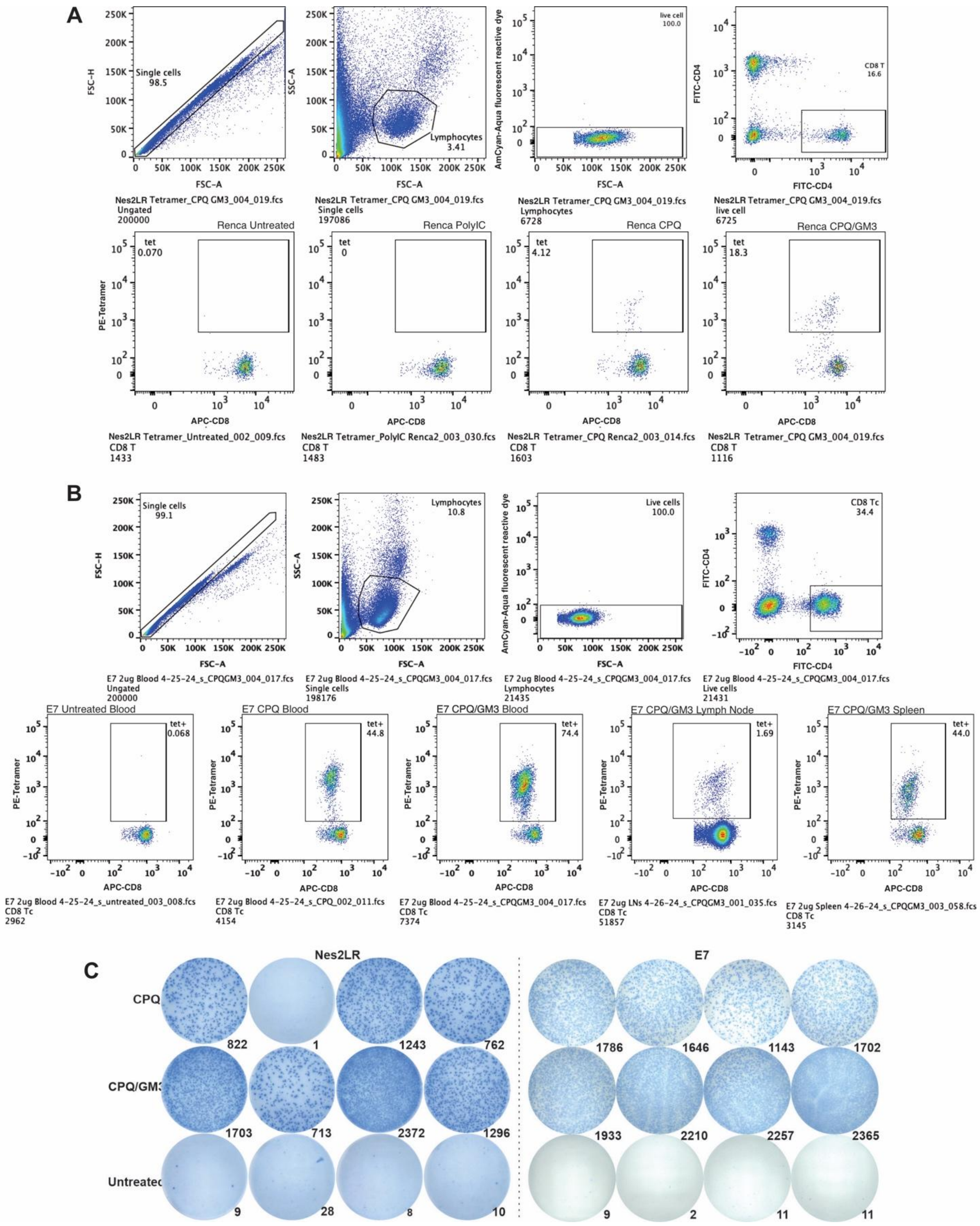

Figure S5. Representative flow cytometry gating strategies and IFN-γ ELISPOT images. A) Representative flow cytometry gating strategy for targeting Nes2LR tetramer⁺ CD8⁺ T cells, **B)** Representative flow cytometry gating strategy for targeting E7 tetramer⁺ CD8⁺ T cells, and **C)** splenocytes IFN-γ ELISPOT well images of Nes2LR and E7 vaccine immunized mice.

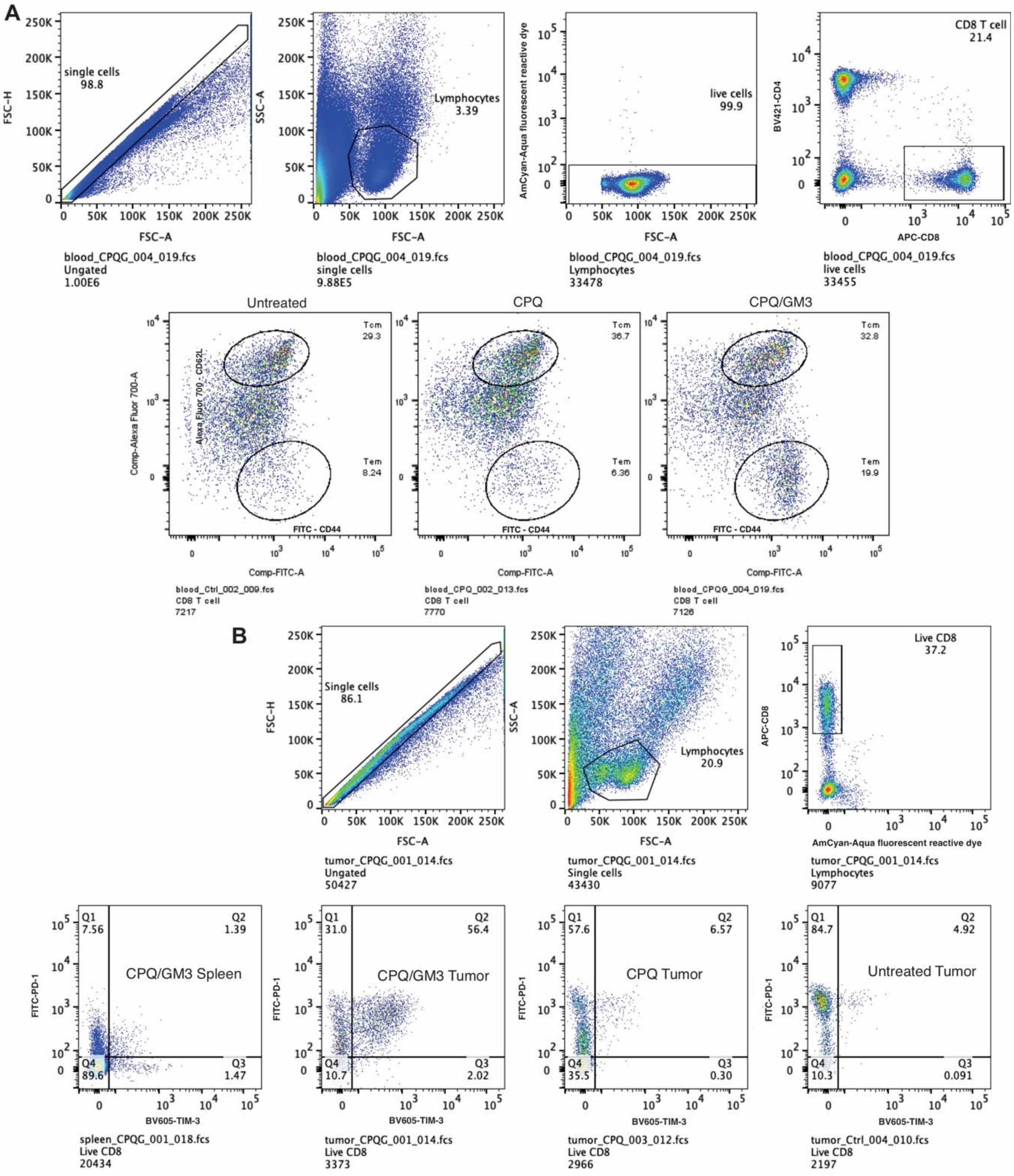

Figure S6. Representative flow cytometry gating strategies. Representative gating strategy and flow cytometry plot for **A)** Tem and Tcm CD8⁺ T cell population tumor-bearing mice; **B)** PD-1 and Tim-3 expression on CD8⁺ T cells.
